# The Relevancy of Data Regarding the Metabolism of Iron to Our Understanding of Deregulated Mechanisms in ALS; Hypotheses and Pitfalls

**DOI:** 10.3389/fnins.2018.01031

**Published:** 2019-01-15

**Authors:** Camille Petillon, Rudolf Hergesheimer, Hervé Puy, Philippe Corcia, Patrick Vourc’h, Christian Andres, Zoubida Karim, Hélène Blasco

**Affiliations:** ^1^Laboratoire de Biochimie, CHRU de Tours, Tours, France; ^2^INSERM, U1253, Université de Tours, Tours, France; ^3^Centre de Recherches sur l’Inflammation, Equipe “Hème, Fer et Maladies Inflammatoires”, UMR 1149/ERL CNRS 8252, Université Paris Diderot Paris 7, UFR de Médecine Site Bichat, Paris, France; ^4^Centre SLA, Service de Neurologie, CHRU de Tours, Tours, France

**Keywords:** iron metabolism, amyotrophic lateral sclerosis, ferritin, biomarkers, ALS

## Abstract

Amyotrophic lateral sclerosis (ALS) is a neurodegenerative disease caused by the loss of motor neurons. Its etiology remains unknown, but several pathophysiological mechanisms are beginning to explain motor neuronal death, as well as oxidative stress. Iron accumulation has been observed in both sporadic and familial forms of ALS, including mouse models. Therefore, the dysregulation of iron metabolism could play a role in the pathological oxidative stress in ALS. Several studies have been undertaken to describe iron-related metabolic markers, in most cases focusing on metabolites in the bloodstream due to few available data in the central nervous system. Reports of accumulation of iron, high serum ferritin, and low serum transferrin levels in ALS patients have encouraged researchers to consider dysregulated iron metabolism as an integral part of ALS pathophysiology. However, it appears complicated to suggest a general mechanism due to the diversity of models and iron markers studied, including the lack of consensus among all of the studies. Regarding clinical study reports, most of them do not take into account confusion biases such as inflammation, renal dysfunction, and nutritional status. Furthermore, the iron regulatory pathways, particularly involving hepcidin, have not been thoroughly explored yet within the pathogenesis of iron overload in ALS. In this sense, it is also essential to explore the relation between iron overload and other ALS-related events, such as neuro-inflammation, protein aggregation, and iron-driven cell death, termed ferroptosis. In this review, we point out limits of the designs of certain studies that may prevent the understanding of the role of iron in ALS and discuss the relevance of the published data regarding the pathogenic impact of iron metabolism deregulation in this disease and the therapeutics targeting this pathway.

## Introduction

Amyotrophic lateral sclerosis (ALS) is a fatal, neurodegenerative disorder characterized by the selective loss of motor neurons (MN) in the spinal cord, brainstem, and cerebral cortex. This leads to progressive paralysis and death by respiratory failure (Hadzhieva et al., 2013). The median survival is three to five years from onset, and the only drug approved for treatment in Europe is riluzole that extends the average patient’s lifespan for only a few more months. ALS is either familial (5–10% of cases) or sporadic (90–95% of cases). Genetic factors including mutations in the *C9orf72*, *SOD1* (Superoxide Dismutase 1), *TARDBP* (Transactive Response DNA-Binding Protein 43), and *FUS* (Fused in Sarcoma) genes could explain nearly 50% of familial cases (van Blitterswijk et al., 2012). Although the specific etiology of sporadic ALS remains unknown, several pathophysiological mechanisms have been described, such as oxidative stress, glutamate-mediated excitotoxicity, impaired axonal transport, mitochondrial dysfunction, and protein aggregation (Shaw, 2005).

Oxidative stress, characterized by reactive oxygen species (ROS), has been suggested to play a major role in the disease, because it has been described in all cases (D’Amico et al., 2013), even in those independent of mutations in SOD1 (corresponding to 80% of familial ALS patients, and most of the cases of sporadic forms). One of the crucial factors associated with the production and presence of ROS is iron, especially via the Fenton reaction, producing highly reactive hydroxyl radicals (Hadzhieva et al., 2013). Furthermore, an iron-dependent mechanism of cell death has recently been described, termed ferroptosis (Dixon et al., 2012). Various reports have suggested a neurodegenerative role of ferroptosis in which the affected neurons exhibit an accumulation of iron and lowered glutathione levels in rodent models (Wu et al., 2018).

Interestingly, perturbed iron regulation has notably been observed in many pre-clinical and clinical studies of ALS (Kwan et al., 2012; Golko-Perez et al., 2017). Determining the etiology of this deregulation, the specific role of iron, whether or not iron is only linked to oxidative stress and, finally, its potential as a diagnostic tool, prognostic marker, or therapeutic target all remain challenges. Studies evaluating iron metabolism in ALS, yet rarely included confusion biases or related these mechanisms to other pathophysiological pathways. In addition, many studies have focused on metabolic markers in the bloodstream, but ALS is mainly a disease of the central nervous system (CNS) and iron metabolism is different in the brain.

Accordingly, we question whether the quantity and the quality of the published data enable us to propose one or several mechanisms linking iron to ALS and to justify their relevance in clinical practice.

We focus this review on the publications concerning the metabolism of iron in the brain, the methods applied to explore iron metabolism in ALS, and on the published data on ALS patients and mutant SOD1 mice. We discuss the role of each marker of iron metabolism evaluated in this pathological context. We also highlight limits of the designs of certain studies that may prevent the understanding of the role of iron in ALS. Lastly, we offer suggestions regarding pitfalls to avoid when determining the role of iron metabolism in this disease’s pathogenesis and discuss the associated therapeutic perspectives.

## Body Iron Metabolism

Body iron metabolism relies on the coordination of several proteins having crucial roles in iron maintenance. Ferritin and transferrin (Tf) are the main carriers of iron in the blood, while proteins such as intracellular iron regulatory proteins (IRPs) and systemic hepcidin are key factors of iron metabolism regulation. Other proteins, including divalent metal transporter-1 (DMT1) and ferroportin (FPN1) in association with ferroxidases such as duodenal cytochrome B (DcytB), hephastin (Hp) and ceruloplasmin (Cp), are involved in the cellular membrane trafficking of iron. Finally, the production of hemoglobin (Hb), myoglobin, and various enzymes (cytochromes, aconitases, etc.) that require iron to function, are the final actors of iron metabolism.

Iron storage and recycling are directed by the liver and the spleen’s macrophages that store excess iron with ferritin. They recycle iron following phagocytosis of senescent red blood cells and degradation of hemoglobin. Released iron is exported by FPN1 then oxidized by Cp to be re-bound to serum Tf for reuse. Iron absorption is mediated by the duodenal epithelial cells, which absorb both heme and non-heme iron from the diet according to the body’s needs. Heme is taken up by specific carrier proteins and is catabolized by heme-oxygenase-1 to release ferrous iron (Fe^2+^). Non-heme ferric iron (Fe^3+^), is reduced to Fe2^+^ by the duodenal Dcytb before uptake by the apically expressed DMT1. Ferrous iron is delivered to the circulation via the basolateral exporter FPN1 in association with the ferroxidase Hp. The complex (Fe^3+^-Tf) is delivered to cells by binding to its receptor, transferrin receptor 1 (TfR1), then the complex is taken up by the cell through the invagination of the membrane, initiating endocytosis. Finally, iron is released from transferrin and is transported out of the endosome by DMT1. In all cells of the body, excess Fe^2+^ is converted to Fe^3+^ by the ferroxidase activity of ferritin H-chain (Ft-H) before storage in the ferritin mineral core (Ft-H and Ft-L) (Hentze et al., 2010).

Hepcidin is a central player in iron homeostasis, acting as a 25 amino-acid mature peptide predominantly expressed in the liver (Nicolas et al., 2001). Hepcidin acts by triggering FPN1 activity and/or protein level and degradation of duodenal DMT1 protein (Nemeth et al., 2004; Brasse-Lagnel et al., 2011; Aschemeyer et al., 2018). Therefore, hepcidin is a hyposideremic factor that reduces both the intestinal absorption of iron and its release by macrophages, hepatocytes, and other cell types. Hepcidin is eliminated in the urine, and small fragments from degradation of 20–25 aa have been found (Park et al., 2001). Hepcidin is expressed by the *HAMP* gene located on chromosome 19 (19q13.1). This gene is upregulated by iron overload, infection, and inflammation. It is downregulated by iron deficiency, hypoxia, anemia, and stimulated erythropoiesis (Hentze et al., 2010).

## Brain Iron Metabolism

**FIGURE 1:**
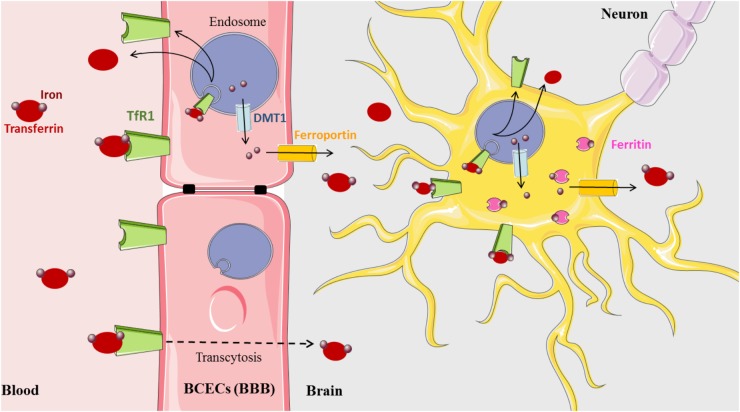
Brain iron metabolism (Medical images: https://smart.servier.com).

Iron is a vital cofactor in many metabolic processes of the CNS, including oxidative phosphorylation, myelin synthesis, neurotransmitter production, and oxygen transport (Crichton et al., 2011). The presence of the blood–brain barrier (BBB) protects the brain from the daily fluctuations of systemic iron and also from iron disorders that affect all peripheral tissues. The precise mechanism by which the BBB mediates iron influx from the blood to the parenchyma of the brain still remains to be uncovered (Figure 1). One hypothesis currently accepted for the transfer of iron across the blood capillary endothelial cells (BCECs) states that Tf-TfR1 transcytosis creates a passive conduit without intracellular processing. However, a new model based on the classical process of iron and experimental data has been recently proposed (Simpson et al., 2015). In this model, iron that enters the CNS is delivered to BCECs by the Tf-TfR complex. Upon internalization into the endosome, iron is released from Tf, exported to the cytosol by DMT1, then exported out of the cell by FPN with the help of Cp ferroxidase expressed at the external cell surface of astrocytes (Jeong and David, 2003; Wu et al., 2004). The *bioGPS* database also shows Cp and FPN transcripts evenly distributed throughout the CNS (BioGPS – your Gene Portal System [Internet], 2018).

Released iron is most likely captured by brain Tf that is synthesized and secreted by oligodendrocytes and the choroid plexus into the brain interstitium. Intriguingly, in this proposed model, evidence of the presence of DMT1 in BCECs is yet to be demonstrated since, not until recently, there are conflicting studies indicating both the presence and absence of DMT1 transcripts and protein (Gunshin et al., 1997; Moos et al., 2006; Skjørringe et al., 2015). However, several arguments promote that in BCECs, the expression level of DMT1 is probably low but that its amount is sufficient to ensure the iron export from the endosome (Skjørringe et al., 2015). A third, alternative explanation of iron delivery involving the uptake of ferritin across the BBB has been proposed, but further support is still necessary to confirm this mechanism (Fisher et al., 2007; Todorich et al., 2011).

The acquisition of iron by brain cells is mediated by different pathways. Neurons express abundant TfR1 and acquire most of their iron via transferrin. Astrocytes do not appear to express TfR1 (Dringen et al., 2007), although this is controversial (Qian et al., 1999). However, they express DMT1 and FPN at the plasma membrane (Wu et al., 2004; Lane et al., 2010). Oligodendrocytes accumulate the most amount of brain iron and are the predominant producers of Tf in the brain (Burdo and Connor, 2003). Transferrin is widely distributed in the CNS but is preferentially expressed in the spinal cord, pre-frontal cortex, and hypothalamus (Mackenzie et al., 2007). Oligodendrocytes also express the ferritin receptor Tim-2, suggestive of an ability to acquire iron from ferritin (Hulet et al., 1999). Most brain cells express cytosolic ferritin for storing excess iron, and relatively high transcript levels are found in the spinal cord, hypothalamus, and pre-frontal cortex; neurons contain the least, while microglia contain the most (Mackenzie et al., 2007; Crichton et al., 2011; Singh et al., 2014).

Despite the high concentration of hepcidin in the liver, recent studies have also revealed the widespread expression of hepcidin in the brain, specifically in the spinal cord, hippocampus, and olfactory bulb (Zechel et al., 2006; Mackenzie et al., 2007; Wang et al., 2008). These findings imply that hepcidin might also be a key regulator in brain iron homeostasis. Hepcidin, similar to its action in the intestine, may regulate the expression of iron transport proteins DMT1 and ferroportin (Puy et al., 2018). This could explain the maintenance of iron balance in neurons and glial cells through the control of their iron uptake and release (Du et al., 2015).

One regulatory protein of brain iron that is currently receiving increased attention is the HFE protein. Mutations in the *HFE* gene are commonly associated with hereditary hemochromatosis in which an iron overload is the consequence of hepcidin deficiency. HFE transcripts in the CNS are relatively concentrated in the spinal cord, temporal and occipital lobes, and the cortex (Mackenzie et al., 2007). The presence of the HFE protein at the interface between the brain and endothelial cells of the microvasculature, and in the cerebrospinal fluid (CSF), may also affect iron content and contribute to iron overload in neurodegenerative disorders. Indeed, HFE mutations have been investigated as risk factors for neurodegenerative disorders (Nandar and Connor, 2011).

## Methods to Study Iron Metabolism

Imaging and biological methods have been utilized on cohorts of patients as tools to study the dysregulation of iron metabolism. In SOD1 transgenic mice and cell models, several approaches, such as concentration measurements, protein expression followed by Western blotting and/or immunofluorescence, and measuring mRNA expression have been employed. Table 1 summarizes the different studies performed to explore iron metabolism in ALS. It is important to notice the significant diversity among models, tissues, and markers studied. For example, cell lines, especially neurons or MN (NSC-34, SH-SY5Y), have been transfected with mutant SOD1 gene constructs in order to attempt to mimic ALS in humans (Schlachetzki et al., 2013). Glial cells are also used in co-culture with neurons, given the hypothesis that ALS is a non-cell-autonomous disease, meaning that multiple cell types can play into the disease’s pathogenesis (Smethurst et al., 2016; Zeineddine et al., 2017). Astrocytes expressing WT or mutant SOD1 have been co-cultured with primary cortical neurons (Kunze et al., 2013). Microglia expressing mutant SOD1 have been co-cultured, as well, with iPSC-derived MN (Frakes et al., 2014). Another example is the co-culture of human adult primary sALS astrocytes and human embryonic stem cell-derived MN (Re et al., 2014).

**Table 1:** Models and iron metabolism markers studied.

Model	Sample	Marker studied	Studies	Workforce
G93A-SOD1 cells	SH-Y5Y human NeurobLastoma cells	DMT1 and TfRl	Hadzhieva et al., 2013	—

SOD1 G93A mouse	Spinal cord	Iron	Lee et al., 2015	4 SOD1 G93A/4 wild-type

SOD1 G37R mouse	Spinal cord	Iron DMT1, TfR1 ferroportin, ceruloplasmin	Jeong et al., 2009	4 SOD1 G37R/ 3 wild-type

SOD 1G93A rats	Serum	Hepcidin	Halon et al., 2014	15 SOD1 G93A/15 wild-type

ALS patients	Serum	Iron, transferrin, ferritin, TSC	Nadjar et al., 2012	629 ALS/297 controls
			Veyrat-Durebex et al., 2014	104 ALS/145 controls
			Goodall et al., 2008	60 ALS/ 44 controls
			Ikeda et al., 2012	92 ALS/ 92 controls
		
		Ferritin	Qureshi et al., 2008	30 ALS/ 30 controls
			Su et al., 2015	138 ALS/152 controls
		
		Pro-hepcidin	Mitchell et al., 2010	30 ALS/ 36 controls
	
	Cerebral cortex	Iron	Oba et al., 1993	15 ALS/49 controls
			Ignjatovic et al., 2013	46 ALS/ 26 controls


*BCECs, blood capillary endothelial cells; BBB, blood–brain barrier; TfR1, receptor transferrin 1; DMT1, divalent metal ion transporter 1.*

Nevertheless, transgenic mice and rats represent the gold standard of preclinical ALS modeling. Rodent models exist for the genetic mutations in *SOD1, TARDBP*, and *FUS* and mouse models for *C9orf72* repeat expansions have also been developed (Chew et al., 2015; Jiang et al., 2016; Liu et al., 2016). In particular, the models in which human genomic mutant SOD1 is overexpressed have been used extensively with the following most studied mutations: SOD1 G93A, SOD1 G37R, and SOD1 G85R (Shaw, 2005). Clinical studies of cohorts of ALS patients also add to the knowledge of iron metabolism.

## Markers of Deregulated Iron Metabolism in ALS

We searched the *Pubmed* database to identify potentially relevant studies on the association between iron metabolism and ALS. We used free text and the medical subject heading (MeSH) terms “amyotrophic lateral sclerosis,” “ALS,” “iron,” “iron metabolism,” “iron accumulation,” “biomarker,” and “iron chelation.” Then, we searched for each marker of iron metabolism (DMT1, TfR1, ferritin, ferroportin, hepcidin, *HFE*,…) AND ALS (OR “amyotrophic lateral sclerosis”).

### Iron Accumulation

Increased iron levels were reported in spinal cord MN in two ALS SOD1-mouse models, SOD1 G93A (Lee et al., 2015) and SOD1 G37R (Jeong et al., 2009), by staining ventral MN with Perl’s (Fe^3+^) and Turnbull’s (Fe^2+^) reactants.

Iron accumulation was also observed in the CNS of ALS patients by magnetic resonance imaging (MRI) (Oba et al., 1993; Ignjatovic et al., 2013). T2-weighted MRI scans were used to detect areas of hypo-intensity (iron deposits) in the cerebral cortex of ALS patients. Likewise, they used Perl’s-reactant for the analyses of postmortem brains and revealed a large amount of ferric iron in the pre-central cortices (Oba et al., 1993). More recently, accumulated iron was detected in multiple brain regions, including the motor cortex, substantia nigra, globus pallidus, red nucleus, and the putamen in ALS patients by quantitative susceptibility mapping (Acosta-Cabronero et al., 2018).

### The Alteration of Iron Transport

A study reported the upregulation of DMT1 and TfR1 mRNAs in G93A-SOD1 cells (SH-SY5Y) (Hadzhieva et al., 2013). In another study, the expression of various proteins involved in iron transport, such as DMT1, TfR1, ferroportin, and ceruloplasmin was evaluated in the spinal cord of SOD1 G37R mice (Jeong et al., 2009). Data at 12 months of age (end stage) showed an increase in the mRNA and protein expression of DMT1, ferroportin, and ceruloplasmin in the cervical spinal cord. Furthermore, double immunofluorescence labeling revealed that in 12-month-old SOD1 G37R transgenic mice the expression of DMT1 was augmented in MN in the ventral horn of the spinal cord. It was also shown that these iron transport markers were more abundant in SOD1 G37R mice at 4 months of age, and mRNA levels were higher in the lumbar region of the spinal cord in the early stages of the disease.

Two independent clinical studies have shown increased values of transferrin saturation (TS) in a cohort of ALS patients and controls (Nadjar et al., 2012; Veyrat-Durebex et al., 2014). However, only the study by Nadjar et al. (2012), performed on a large ALS population (694 patients), thus presenting higher statistical power (Veyrat-Durebex et al., 2014), showed a significant decrease in serum transferrin levels in ALS subjects. Interestingly, it was also demonstrated that low serum transferrin concentrations were associated with a higher loss in body weight at diagnosis in ALS patients (Veyrat-Durebex et al., 2014), which could link iron metabolism to nutritional status – largely associated with energetic metabolism in ALS.

### Increased Serum Ferritin

Serum ferritin is also increased in ALS patients (Goodall et al., 2008; Qureshi et al., 2008; Su et al., 2015). Six case-control studies published between 2008 and 2015 investigating the possible relationship between ALS patients and their susceptibility to elevated serum ferritin levels were included in a meta-analysis (Hu et al., 2016), including 1053 cases of ALS and 760 healthy control cases from the United Kingdom, United States, France, and Japan. The serum ferritin concentrations in ALS were statistically higher compared to healthy controls (*p* < 0.00001). The limitations revealed by this meta-analysis were a high heterogeneity (*p* = 0.03; I^2^ = 50%) and a publisher bias.

### Alteration of *HFE* Gene and Hepcidin Alteration

A meta-analysis assessing the roles of the H63D and C282Y variants of the *HFE* gene in ALS from 14 observational studies illustrated a significant association of the C282Y variant to the disease, meaning a decreased risk for ALS, while confirming that the H63D variant showed no relevant association (Li et al., 2014). In fact, hemochromatosis linked to mutations in the *HFE* gene has been attributed solely to the homozygous C282Y variant. The results of this meta-analysis imply that the C282Y *HFE* variant could be a protective factor against ALS, which is *a priori* surprising given the physiopathology of ALS, in which iron is expected to play a toxic role.

In one report, hepcidin plasma concentrations were measured in SOD1 transgenic rats (G93A) at 12 weeks (asymptomatic, ALS stage I), 21 weeks (disease onset, ALS stage II) and 24 weeks (end-stage disease, ALS stage III) of age, and were significantly increased in the blood of ALS II and III animals (Halon et al., 2014). On the other hand, the plasma concentrations of pro-hepcidin, the precursor to hepcidin, were not significantly altered in a cohort of 29 ALS patients (from nine to 28 months post-diagnosis) compared to control group. Nevertheless, there was a positive correlation between pro-hepcidin and IL-6 levels in ALS patients compared to controls, suggesting exacerbated iron release by macrophages and inflammation in ALS patients (Mitchell et al., 2010).

## Can We Integrate Published Data to Suggest a Role of Iron Deregulation in ALS?

Regarding the diversity of models and the methodology used, as well as the studied markers, the findings with respect to iron metabolism seem heterogeneous. It appears complicated to suggest a global mechanism of dysregulation. Accumulation of iron, higher serum ferritin, and lower serum transferrin levels in ALS patients have clearly encouraged researchers to consider iron deregulation as an essential mechanism in ALS. The critical review of the literature led us to identify the limits preventing the understanding of iron metabolism disturbance in ALS, and more specific findings in the context of ALS must be provided.

## Pitfalls to Avoid When Studying Iron Metabolism in ALS

### Types of Models

The lack of the complex interplay between MN and their surrounding environment is the precise problem that we face with the published data on cellular models, encouraging us to turn to other models. Although SOD1 mutations are rare in sALS, there are phenotypic similarities with fALS, and it has been assumed that studying the familial form could provide insight into the sporadic form. However, the validity of these assumptions has been questioned (Clerc et al., 2016) and, even if clinical manifestations are not so different, the pathophysiological mechanisms are obviously distinct. For instance, TDP-43 aggregates were not observed in patients suffering from ALS forms associated with SOD1 mutations (Mackenzie et al., 2007), whereas they were in a SOD1^G93A^ mouse model (Jeon et al., 2018). Due to this discrepancy between postmortem patient and mouse tissues, one should consider the TDP-43 aggregation to be unrelated to the SOD1 ALS pathology if this artifactual aggregation is unavoidable in SOD1 mouse models. Moreover, exploring iron metabolism closely linked with the consequence of SOD1 activity, i.e., production of ROS, seems biased. So, this model that is subjected to oxidative stress may not fit with the investigation of iron metabolism. In addition, the relatively rapid neurodegeneration that occurs in these mice can reduce the quantity of iron in the affected neurons, potentially confounding the interpretation of the corresponding results. Altogether, we are aware that SOD1 mouse models remain valid for the study of certain aspects of ALS pathogenesis, but the scope of the conclusions must be limited in relation to the restraints of this model, especially in the exploration of iron metabolism.

### Localization of Iron Metabolism Exploration

Data reporting the main genetic loci involved in iron accumulation would be the first step to the understanding of the role of iron in ALS. Another study, in fact, showed that inducing inflammation with intravenous injections of lipopolysaccharide (LPS) in mice led to hepcidin expression in the cortex, hippocampus, and striatum (Wang et al., 2008). In fact, hepcidin along with the other iron regulators are shown to be expressed in the spinal cord and cortex, the two main areas affected by ALS. Therefore, investigating their behavior in these tissues in ALS models could yield insight regarding iron accumulation hot spots. Published data on mouse models have revealed that the spinal cord is especially affected with increased expression of iron transporters (DMT1, ferroportin, and TfR1). Much attention has been turned toward ALS patients’ brains, but incomplete data prevent us from drawing conclusions in humans. Although there is limited data for factors of iron metabolism in the CSF of ALS patients, data from MRI and postmortem brain exploration have presented interesting information to locate the main regions of accumulation.

In addition, the identification of cells types is also essential to integrate all of these findings. Some researchers have highlighted the role of glia (Jeong et al., 2009) by observing an increased cytosolic ferritin expression not identified in MN, suggesting that neurons and glia accumulate iron via different mechanisms.

Finally, it is necessary to be aware of the differences in the expression levels between mice and humans, since murine models are the “gold standard” in studying ALS. Indeed, the differences in distribution and expression levels pose limitations on the conclusions inferred from these models (Supplementary Table S1). Even though certain regulators exhibit differential distributions and expression levels in the mouse CNS compared to that of humans, mouse microglia consistently show expression for most, if not all, the regulators mentioned here. Because cells other than neurons can also become affected in ALS and that microglia have quite an unequivocal relationship with iron-driven inflammation, paying attention to the levels of these iron regulators in the microglia of murine ALS models could increase the relevancy and decrease the limitations of murine models.

### Extrapolation From Liver to the Brain

The interaction between the liver and the brain regarding iron metabolism must be taken into account, but it is difficult to extrapolate from the published data. Indeed, most of the factors of iron metabolism have not yet been studied in the brain, and we cannot deduce a mechanism from their activity in other organs. For example, it was suggested that the HFE C282Y variant could be a potentially protective factor for ALS. But, it is difficult to extrapolate the role of HFE from the liver to the brain, given that ALS is not a disease primarily affecting the liverapart from the manifestation of a fatty liver (Dupuis et al., 2008). Similarly, it could be misleading to compare the levels of metabolic regulators of iron in the serum and the brain, since the main carriers and regulatory processes are not fully understood in healthy persons and even less so in ALS patients.

## Relevant Parameters to Include in Iron Metabolism Exploration in ALS Patients

### Dynamic Variation of Iron Metabolism

Importantly, a multi-factorial disease, such as ALS, is characterized by regulation and counter-regulation, which is dependent on the progress of the disease and on other pathophysiological mechanisms. Information regarding the relation between iron metabolism and disease progression would help the integration of iron’s role in the pathogenesis. One does, therefore, wonder if iron accumulation in the CNS of ALS patients is a factor for poor prognosis and if increasing concentrations of iron might cause the disease to progress more quickly over time. However, very few data on iron concentrations in the brain of ALS patients are available, and even fewer about variations in these concentrations throughout the progression of the disease. Nadjar et al. (2012) showed that high levels of serum ferritin (>156 μg/L), were significantly associated with a reduced survival time. ALS patients with low levels of ferritin lived around 300 days longer than patients with high levels (*p* < 0,04). But, serum ferritin does not necessarily reflect the iron accumulation in the CNS.

The pathophysiological mechanisms that could explain why ALS patients have increased iron body storage and how that is responsible for a decreased survival is, at present, unknown.

With respect to the largely unknown mechanism of regulation and/or rebound effect, very little can be concluded for a general mechanism of iron metabolism in ALS, such as with data concerning hepcidin that has been clearly understudied and whose studies yield contradictory outcomes.

### Confounding Factors for the Interpretation of Iron Metabolism Status

Regarding clinical studies, the first issue is the heterogeneity among ALS patients, since the site of onset, the form of ALS, the evolution, and genetic mutations add and multiply the diversity among patients. To add to this expected and known limit of all cohort studies of ALS, some biological concerns are often overlooked. Several parameters that can disrupt iron metabolism are not taken into account. For example, inflammation, renal dysfunction, and nutritional status are commonly overlooked. Since serum ferritin is an acute-phase reactive protein whose concentration can rise in the presence of acute or chronic inflammation, it might fluctuate depending on the inflammatory status of ALS patients (Hu et al., 2016). Also, CRP concentrations were not measured in all the clinical studies (Qureshi et al., 2008; Ikeda et al., 2012; Nadjar et al., 2012). This was taken into account in the study by Veyrat-Durebex et al. (2014), and CRP values were not significantly different between patients and controls. In Goodall et al. (2008), CRP and ceruloplasmin levels were both negatively correlated with iron and iron saturation in ALS patients showing that iron metabolism disturbance was not due to inflammation.

Kidney dysfunction may alter some parameters of iron metabolism (Maras et al., 2015). The age at onset of ALS [between 50 and 60 years (Shaw, 2005)] could suggest that kidney function starts to be altered, but this data has not been directly reported; no ideal marker has been identified or used in these cases, and clinicians are hesitant to insist on kidney function exploration. Unfortunately, creatinine is not a very reliable marker of kidney function in the context of ALS due to muscular atrophy (Corbett et al., 1982), leading to the mis-evaluation of the glomerular filtration rate (eGF). Veyrat-Durebex et al. (2014) reported a creatinine value, used in the MDRD (modified diet in renal disease) equation, to estimate the glomerular filtration rate. The estimated eGFR was higher (*p* < 0.0001) in ALS patients than in controls, showing not that renal function was better but that creatinine levels were low probably due to muscular atrophy and that the role of potential defective renal elimination on differences in iron metabolism cannot be explored by this approach (Veyrat-Durebex et al., 2014). The evaluation of creatinine values after taking into account body composition (impedancemetry) or using other markers of renal function (cystatin C, iohexol, etc.) may help with the interpretation of iron metabolism, independent of muscle atrophy and renal elimination. Similarly, nutritional factors essential to ALS have to be included in iron metabolism interpretation, as it may completely influence the results, either due to disease or management. For example, it has been illustrated that low serum transferrin concentrations are associated with a higher body weight loss at the time of diagnosis in ALS patients (Veyrat-Durebex et al., 2014). Further investigations on nutritional care in ALS patients will be necessary.

We expect promising and complementary findings from CSF studies, but few data has been published to date (Goodall et al., 2008).

## Therapeutic Prospects

The pathogenic impact of iron metabolism deregulation in ALS has been further supported by the partially protective effects of iron chelators in ALS mouse models (Hadzhieva et al., 2014). In recent years, various iron chelators have demonstrated a prolonged survival of SOD1 transgenic mice. A classic iron chelator, desferrioxamine, previously studied in models of neurodegenerative diseases, such as Huntington’s disease or Parkinson’s disease (Hilditch-Maguire et al., 2000; Zhang et al., 2005), as well as the new iron chelators 5-[4-(2-hydroxyethyl) piperazine-1-ylmethyl]-8- hydroxyquinoline (VK-28) and 5-(*N*-methyl-*N*-propargylaminomethyl)-8-hydroxyquinoline (M30), have also been studied in models of ALS disease (Wang et al., 2011).

Iron chelators significantly delay disease onset (6–12 days on average, *p* < 0.05), extend the lifespan (8–13 days on average, *p* < 0.05), and reduce spinal cord motor neuron loss in ALS SOD1 transgenic mice. These chelators are able to prevent ferroptosis, as well (Masaldan et al., 2018). In support of this, a mouse model of aggravated ferroptosis displayed ALS features, such as intense muscular atrophy, rapid paralysis, and ferroptotic neurodegeneration of the motor neurons of the spinal cord (Chen et al., 2015; Masaldan et al., 2018). Thus, the therapeutic effects of iron chelation in SOD1 mice seem to partly stem from the suppression of ferroptosis, which could result from iron accumulation. Besides these findings, VK-28 and M30 reduced ROS generation and inhibited activation of glial cells in the spinal cord (Wang et al., 2011).

In both a SOD1 murine model of fALS and sALS, a low dose of deferiprone, another classic iron chelator, was associated with a decrease in pathological iron accumulation in the central motor pathways (Moreau et al., 2018).

In ALS patients, iron chelation could reduce iron accumulation and the related excess of oxidative stress in the motor pathways. Classical iron chelation could induce systemic iron depletion. For this reason, the safety and efficacy of conservative iron chelation (chelation with a low risk of iron depletion) was assessed in a single-center, single-arm, 12-month-long pilot clinical trial (NCT02164253). After 12 months of treatment with deferiprone, none of the 23 ALS patients enrolled in the trial displayed signs of anemia. The decreases in the ALS Functional Rating Scale (ALSFRS-R) and in the body mass index were significantly smaller in the first 3 months of deferiprone treatment than in the parallel 3-month treatment-free period (Moreau et al., 2018). A significant decrease in iron concentration, shown by MRI, was observed in the cervical spinal cord and the motor cortex while not in areas outside the motor system (the cerebellum and the occipital cortex) (Moreau et al., 2018).

Currently, the efficacy of deferiprone in ALS patients is being evaluated in a randomized, double-blind, placebo-controlled, multi-center French study (FAIR-ALS II) with an estimated enrollment of 210 participants and a 12-month period (NCT03293069).

Evidence has revealed that the neuroprotection of iron chelation may result from the attenuation of iron-related oxidative stress, iron accumulation, and ferroptosis. However, we have little perspective on the long term regarding the effects of these iron chelators, and we are not yet certain about their precise mechanism of action. It could very well be that the chelation treatment brings about a long-term effect; it could also simply be temporarily effective, requiring life-long treatment. Moreover, to our knowledge no study has determined the exact specificity of these chelators for iron. We question to which extent one can attribute the therapeutic effect of iron chelators in animal models to the interaction with iron. Therefore, future studies should pay attention to the concentrations of other heavy metals besides iron in order to better assess the overall effect of the chelator on the cell.

## Conclusion

Since iron heavily participates in ROS production and has been seen to accumulate in the context of ALS, we have focused this review on the published findings concerning iron metabolism in this disease. Future studies will need to address the experimental limitations that we have acknowledged in this review. The limitations in the murine models originate from the experimental bias projected from the use of pathogenic SOD1. Because this condition primarily generates ROS, the conclusions relating iron metabolic regulators drawn from these studies are confined to the ALS cases in which SOD1 is mutated, which is only a minute proportion of both familial and sporadic cases. Therefore, it would be of particular interest to compare the effects on iron regulation between the SOD1 models and another type, such as TDP-43 models.

Other limitations in clinical studies analyzing the metabolic alterations of iron stem from the fact that many overlook the roles of the inflammatory status, renal function, and nutritional status. These factors become very important when considering therapeutic strategies relying on iron chelation. The clinical trials attempt to employ chelators as an early form of treatment but, even though patients show a considerable retention in body mass and a decrease in iron levels in the spinal cord (Wang et al., 2011), this seems only temporary. Furthermore, studies on patients do not necessarily contain a homogeneous cohort of people affected by SOD1-driven ALS, unlike SOD1 murine models. This confounds the interpretations of iron metabolism, since the sub-type of ALS is not controlled. However, these clinical limitations might be nearly impossible to avoid. Therefore, while complementing treatment with nutritional maintenance and keeping track of kidney and inflammatory status, the chelation-guided therapy could be prolonged and possibly more applicable to the general population of ALS patients. Indeed, there appears to be a global dysregulation of iron in ALS cases, and we must gain a more complete understanding of the causes and consequences.

## Author Contributions

CP and RH wrote most of the sections of the manuscript. CP did the bibliography and the literature review on iron in ALS. ZK wrote Section “Body Iron Metabolism” and completed Section “Brain Iron Metabolism”. RH did the article proofreading (English). All authors contributed to manuscript revision, read and approved the submitted version.

## Conflict of Interest Statement

The authors declare that the research was conducted in the absence of any commercial or financial relationships that could be construed as a potential conflict of interest.
