# Supplementary material for: The Relevancy of Data Regarding the Metabolism of Iron to Our Understanding of Deregulated Mechanisms in ALS; Hypotheses and Pitfalls
Source: Front Neurosci. 2019 Jan 15;12:1031. doi: 10.3389/fnins.2018.01031 (PMC6341213; doi:10.3389/fnins.2018.01031)
Supplement: Supplementary file 1 [file Data_Sheet_1.docx]

***Table S1* Differential Expression of Iron Regulators Between Human and Mouse**

The values represent the absolute fluorescence intensities documented on *biogps.com* (19), corresponding to the hybridization of the mRNA transcripts to the microarray chip. The differential expression between mice and humans for a given tissue and regulator is deduced from the comparison of the relative intensities of each organism (i.e. hepcidin is expressed the most in the liver in both species because both organisms show the highest values in that tissue). Each microarray probe set is given here for human and mouse, respectively: hepcidin – 220491_at/1419197_x_at; ceruloplasmin – 204846_at/1417495_x_at; HFE –206086_x_at/1422645_at; DMT1 – 203125_x_at/1452078_a_at; ferroportin – gnf1h00171_at/1448566_at; transferrin –220109_at/1425546_a_at; TfR1 – 207332_s_at/1422966_a_at

| **organ/tissue** | **organism** | **hepcidin** | **ceruloplasmin** | **HFE** | **DMT1** | **ferroportin** | **transferrin** | **TfR1** |
| --- | --- | --- | --- | --- | --- | --- | --- | --- |
| whole blood | human  mouse | 16.25  N/A | 8.55  N/A | 8.55  N/A | 4.05  N/A | 62.30  N/A | 11.60  N/A | 46.65  N/A |
| liver | human  mouse | 5,564.60  11,641.33 | 14.55  15,164.65 | 10.00  1,352.53 | 4.80  42.75 | 24.10  24.10 | 22.70  48,401.42 | 17.15  132.63 |
| whole brain | human  mouse | 16.60  N/A | 6.25  N/A | 6.65  N/A | 3.20  N/A | 24.20  N/A | 7.85  N/A | 42.35  N/A |
| cortex | human  mouse | 35.25  4.86 | 9.95  63.33 | 9.70  8.47 | 4.70  39.52 | 23.15  9.75 | 12.20  2,920.10 | 247.40  131.14 |
| spinal cord | human  mouse | 50.85  4.86 | 8.85  39.01 | 8.20  31.22 | 3.90  30.01 | 24.55  9.62 | 17.70  24,310.06 | 74.10  139.09 |
| microglia | human  mouse | N/A  4.99 | N/A  2,109.85 | N/A  508.20 | N/A  778.51 | N/A  290.37 | N/A  3,840.94 | N/A  1,228.75 |
